# Injection drug use practices and HIV infection among people who inject drugs in Kigali, Rwanda: a cross-sectional study

**DOI:** 10.1186/s12954-021-00579-0

**Published:** 2021-12-15

**Authors:** Jean Olivier Twahirwa Rwema, Vianney Nizeyimana, Neia M. Prata, Nneoma E. Okonkwo, Amelia A. Mazzei, Sulemani Muhirwa, Athanase Rukundo, Lisa Lucas, Audace Niyigena, Jean Damascene Makuza, Chris Beyrer, Stefan D. Baral, Aflodis Kagaba

**Affiliations:** 1 Department of Epidemiology, Key Populations Program, Center for Public Health and Human Rights, Johns Hopkins Bloomberg School of Public Health, 615 N Wolfe Street E 7133, Baltimore, MD 21205 USA; 2 Health Development Initiative, Kigali, Rwanda; 3 School of Medicine, Johns Hopkins University, Baltimore, MD USA; 4 Emory University School of Medicine, Atlanta, GA USA; 5 Projet San Francisco, Kigali, Rwanda; 6 Département de Psychiatrie, Service d’addictologie, Hôpitaux Universitaire de Genève, Geneva, Switzerland; 7 School of Population and Public Health, University of British Columbia, Vancouver, BC Canada; 8 BC Center for Disease Control, Vancouver, BC Canada; 9 Rwanda Biomedical Center, Kigali, Rwanda

**Keywords:** People who inject drugs, HIV, Injection drug use, Kigali, Rwanda

## Abstract

**Background:**

In Rwanda, epidemiological data characterizing people who inject drugs (PWID) and their burden of HIV are limited. We examined injection drug use (IDU) history and practices, and HIV infection in a sample of PWID in Kigali.

**Methods:**

From October 2019 to February 2020, 307 PWID aged ≥ 18 were enrolled in a cross-sectional study using convenience sampling in Kigali. Participants completed interviewer-administered questionnaires on IDU history and practices and HIV testing. We used Poisson regression with robust variance estimation to assess IDU practices associated with HIV infection and assessed factors associated with needle sharing in the six months preceding the study.

**Results:**

The median age was 28 years (IQR 24–31); 81% (251) were males. Female PWID were more likely to report recent IDU initiation, selling sex for drugs, and to have been injected by a sex partner (*p* < 0.05). In the prior six months, heroin was the primary drug of choice for 99% (303) of participants, with cocaine and methamphetamine also reported by 10% (31/307) and 4% (12/307), respectively. In total, 91% (280/307) of participants reported ever sharing needles in their lifetime and 43% (133) knew someone who died from a drug-related overdose. HIV prevalence was 9.5% (95% CI 8.7–9.3). Sharing needles at least half of the time in the previous six months was positively associated with HIV infection (adjusted prevalence ratio (aPR) 2.67; 95% CI 1.23–5.78). Overall, 31% (94/307) shared needles and 33% (103/307) reused needles in the prior six months. Female PWID were more likely to share needles compared to males (aPR 1.68; 95% CI 1.09–2.59). Additionally, bisexual PWID (aPR 1.68; 95% CI 1.09–2.59), those who shared needles at the first injection (aPR 2.18; 95% CI 1.59–2.99), reused needles recently (aPR 2.27; 95% CI 1.51–3.43) and shared other drug paraphernalia (aPR 3.56; 95% CI 2.19–5.81) were more likely to report recent needle sharing.

**Conclusion:**

HIV infection was common in this study. The high prevalence of needle reuse and sharing practices highlights significant risks for onward transmission and acquisition of HIV and viral hepatitis. These data highlight the urgent need for PWID-focused harm reduction services in Rwanda, including syringe services programs, safe injection education, naloxone distribution, and substance use disorder treatment programs and optimizing these services to the varied needs of people who use drugs in Rwanda.

## Introduction

Globally, 15 million people report injection drug use (IDU), with increasing numbers in countries across sub-Saharan Africa (SSA) [1, 2]. Estimates of IDU range from 0.13 to 0.62% among the general adult population in SSA [1]. IDU is associated with an increased risk of acquiring HIV and other sexually transmitted infections (STIs), hepatitis B (HBV), hepatitis C (HCV), and other blood-borne infections (OBBI), and is associated with increased burden of mental health disorders [2, 3]. The high risk of HIV among PWID can be explained by individual factors like needle sharing, and structural factors including stigma and discrimination, criminalization, high exposure to human rights abuses; and a paucity of harm reduction programs [4, 5]. These structural-level factors limit PWID engagement in prevention and treatment services, resulting in PWID having poorer HIV outcomes compared to other adults [6]. PWID additionally face significant social and economic challenges, as often demonstrated by their high burdens of homelessness, unstable housing, and incarceration rates [1]. Finally, PWID have overlapping sexual risks with high levels of inconsistent condom use and sex work [7].

While the needs of PWID have been widely explored and documented in high-income countries, limited data exist for countries across SSA [1, 8]. However, a review of IDU in six African countries found a high prevalence of high-risk behaviors including needle sharing and inconsistent condom use among PWID [9]. In addition to the paucity of evidence to guide programming, few PWID-centered programs exist [8]. A systematic review of interventions to prevent and manage HIV and HCV among PWID revealed that while more countries in SSA are studying drug use, few countries have increased the implementation of PWID-focused programs over the last decade (2 to 7 for syringe programs, 4 to 8 for opioid substitution therapy, and even fewer for other interventions) [8]. Thus, more research among PWID is needed to guide implementation across SSA.

In Rwanda, HIV prevalence among reproductive aged adults has stabilized at 3% for the last decade, with the country being one of the few to achieve the UNAIDS 90-90-90 HIV elimination targets by 2020 [10]. However, similar progress has yet to be seen among key populations (KPs). Recent studies have demonstrated a high prevalence of HIV and other STIs and programmatic gaps among KPs including Men who Sex with Men, Transgender Women, and Female Sex Workers [11–13]. Moreover, PWID have been the most overlooked in HIV programming and research in Rwanda. Rwanda’s HIV guidelines have a minimum package of services for both MSM and FSW, but no such program exists for PWID [14]. This lack of PWID-focused national programming, coupled with the criminalization of drug use, further complicates programming in Rwanda [10]. Thus, understanding and addressing the needs of PWID in Rwanda is a public health and human rights imperative.

We conducted this study to inform future research and programming for PWID in Rwanda. The aim of this paper was to estimate the burden of HIV infection and of IDU practices among PWID in Kigali.

## Methods

### Study context, procedures, and population

This was a cross-sectional mixed methods study implemented by Health Development Initiative (HDI), a local nongovernmental organization working with KPs in Rwanda. This study leveraged qualitative and quantitative methods to guide implementation of health programs for PWID in Rwanda. The specific objectives were to collect data on sociodemographic characteristics and IDU practices among PWID, to characterize the PWID population, and to provide an estimate of the HIV prevalence among PWID in Kigali.

Given the lack of a sampling frame and nonexistence of reliable epidemiological or program data to guide probability sampling methods for PWID, study participants were recruited through purposive and convenience sampling in Kigali city from October 2019 to February 2020. Initial participants were recruited from clients who use HDI clinical services in Kigali city. HDI offers a range of clinical services to members of different KPs. Clients who reported injecting drugs recently, able, and willing to provide informed consent were recruited into the study. Upon recruitment, these participants were trained and encouraged to recruit their own peers who injected drugs to the study. However, contrary to respondent-driven sampling, there was no maximum number of participants that individuals could recruit. Further community outreach was conducted by HDI community liaisons to recruit more PWID from all three districts of Kigali: Nyarugenge, Kicukiro, and Gasabo. Eligible participants were at least 18 years old, had injected drugs in the six months preceding the study, agreed to be tested for HIV, and gave written consent to participate in the study. The study was approved by the Rwanda National Ethics Committee N: 027/RNEC/2020.

### Data collection

Data collection was conducted in two HDI sites in Kigali city. After signing the informed consent form, participants underwent structured face-to-face interviews conducted by trained data collectors and biological testing for HIV. The questionnaire comprised of questions related to sociodemographic characteristics and IDU history and practices in the months preceding the study. Participants were also asked about their need for, and access to, substance use disorder treatment programs. No additional personal identifying information was collected other than the signature on the written informed consent.

We performed HIV rapid testing for all participants who gave consent, per the national guidelines [14]. The screening test was Alere HIV Combo-Determine (Alere, Inc, Waltham, MA), and the confirmatory test was HIV1/2 STAT-PAK (Medford, NY, USA). Participants with a prior documented HIV diagnosis in a health facility were not retested. Participants who were newly found to be living with HIV were referred to a healthcare facility to initiate antiretroviral treatment (ART) and for further medical management. Participants also received information on centers providing substance use disorder treatment in Rwanda. Upon completion of study procedures, participants received 2000 Frw as transport reimbursement.

### Outcome assessment

The primary outcome was prevalent HIV infection. HIV-positive status was based on testing conducted in the study or previously medically confirmed HIV-positive status. The second outcome was self-reported recent needle sharing (i.e., six months prior to study enrollment).

### Other variables of interest

#### Sociodemographic characteristics

Sociodemographic characteristics included age, sex assigned at birth, education, occupation, and sexual orientation.

Age was collected as a continuous variable but categorized into three groups for analytical purposes: 18–24, 25–34, and  ≥35 years. Sex was defined as female or male based on sex assignment at birth. Sexual orientation was self-reported and categorized as heterosexual, homosexual, and bisexual. Education and occupation were analyzed as categorical variables with three groups each (Table 1).

**Table 1:** Baseline characteristics of PWID in Kigali, Rwanda, N:307

Characteristic	*N*	%	Not living with HIV *N* (%)	Living with HIV *N* (%)
Age in years*				
18–24	83	27.2	80 (96.4)	3 (3.6)
25–34	188	61.6	167 (88.8)	21 (11.2)
≥35	34	11.2	30 (88.2)	4 (11.8)
Biological sex				
Female	56	18.2	48 (85.7)	8 (14.3)
Male	251	81.8	230 (91.6)	21 (8.4)
Education				
Primary education or less	47	15.3	40 (85.1)	7 (14.9)
Some secondary education	79	25.7	76 (96.2)	3 (3.8)
Completed secondary or above	181	58.9	162 (89.5)	19 (10.5)
Marital status**				
Single	252	82.9	232 (92.1)	20 (7.9)
Cohabitating/Married	33	10.8	31 (93.9)	2 (6.1)
Divorced/Separated/Widow	19	6.3	12 (63.2)	7 (36.8)
Occupation				
Unemployed	169	55	154 (91.1)	15 (8.9)
Student	10	3.3	10 (100)	0 (0)
Part time/Full time employee	128	41.7	114 (89.1)	14 (10.9)
Self-reported sexual orientation***				
Heterosexual	215	70.3	198 (92.1)	17 (7.9)
Homosexual	35	11.4	31 (88.6)	4 (11.4)
Bisexual	56	18.3	48 (85.7)	8 (14.3)

* Data missing for n=2; ** Data missing for n=3; *** Data missing for n=1

#### IDU history and practices, access to treatment, and other behaviors

Participants were asked the age at which they first injected drugs, the drug they first injected, source of the drug, and whether they shared needles during their first drug injection. Overall injection history was assessed by estimating the duration of IDU for each participant and lifetime needle sharing history. Participants were also asked if they knew anyone who had died from a drug overdose. Participants were asked detailed questions about their primary drug of injection, other drugs used, and frequency of drug injection in the prior six months. To assess injection frequency, participants were asked the number of times they injected per day, week, month, or year. Frequency of needle sharing, sharing of other injection materials, and needle reuse were assessed using 5-item scales ranging from never, rarely, half the time, most of the time, and always. Information on injection partnerships (i.e., sex partners, friends, relatives, drug dealer, strangers, or other) and history of exchanging sex for drugs were also captured. Participants were asked about their knowledge and use of substance use disorder treatment programs in Rwanda (Table 2). Finally, participants were asked questions assessing their HIV knowledge, HIV testing history, and condom use during sex.

**Table 2:** Injecting drug use history and practices among PWID in Kigali, Rwanda, N:307

Variable	*N*	%	Female *N* (%)	Male *N* (%)	*p* value
*First drug injection*					
Age at first injection					
Less than 18	53	17.3	5 (8.9)	48 (19.1)	0.187
18–24	132	43	27 (48.2)	105 (41.8)	
> 25	122	39.7	24 (42.9)	98 (39.1)	
Drug injected the first time					
Heroin	304	99.1	55 (98.2)	249 (99.2)	0.454
Cocaine	2	0.6	1 (1.8)	1 (0.4)	
Methamphetamine	1	0.3	0 (0)	1 (0.4)	
Person who performed the injection the first time					
Self	134	43.7	21 (37.5)	113 (45.1)	**0.0001**
Friend	146	47.5	17 (30.4)	129 (51.4)	
Sex partner	20	6.5	16 (28.6)	4 (1.6)	
Other	7	2.3	2 (3.5)	5 (1.9)	
Needle sharing the first time					
No	234	76.2	43 (76.8)	191 (76.1)	0.913
Yes	73	23.8	13 (23.2)	60 (23.9)	
Source of drug the first time					
Bought them from someone	259	84.4	38 (67.8)	221 (88.1)	**0.001**
Received them for free	41	13.4	16 (28.6)	25 (9.9)	
Traded them for sex	5	1.6	2 (3.6)	3 (1.2)	
Refused to answer	2	0.6	0 (0)	2 (0.8)	
*Injecting drug history*					
Duration of drug injection*					
Less than 3	129	42.3	32 (58.2)	96 (38.6)	**0.007**
4–5 years	105	34.4	18 (32.7)	87 (34.9)	
Over 5	71	23.3	5 (9.1)	66 (26.5)	
Needle sharing history					
Never shared needles	27	8.8	6 (10.7)	21 (8.4)	0.575
Ever shared needles	280	91.2	50 (89.3)	230 (91.6)	
Used a Drug combination					
No	212	69.5	45 (83.3)	167 (66.5)	**0.015**
Yes	93	30.5	9 (16.7)	84 (33.5)	
Needle sharing in the previous six months					
Never	213	69.4	39 (69.6)	174 (69.4)	0.285
Rarely	60	19.5	8 (14.3)	52 (20.7)	
Half of the time or more	34	11.1	9 (16.1)	25 (9.9)	
Needle reuse in the previous six months					
Never	204	66.5	45 (80.4)	159 (63.4)	**0.05**
Rarely	75	24.4	8 (14.3)	67 (26.7)	
Half of the time or more	28	9.1	3 (5.3)	25 (9.9)	
Selling sex for Drugs in the previous six months*					
No	204	68.5	22 (39.3)	182 (75.2)	**0.0001**
Yes	94	31.5	34 (60.7)	60 (24.8)	
*Need and access to substance use disorders treatment programs*					
Tried to reduce or quit drug consumption in the previous 6 months***					
No	122	39.9	23 (41.1)	99 (39.6)	0.839
Yes	184	60.1	33 (58.9)	151 (60.4)	
Aware of any Drug addiction treatment programs					
No	205	66.7	38 (67.9)	167 (66.6)	0.285
Yes	45	14.7	11 (19.6)	34 (13.5)	
Don't know	57	18.6	7 (12.5)	50 (19.9)	
Drug Addiction treatment in the previous six months****					
No	290	95.4	54 (96.4)	236 (95.2)	0.683
Yes	14	4.6	2 (3.6)	12 (4..8)	
Unable to access Drug Addiction treatment in the previous six months					
No	259	84.4	49 (87.5)	210 (83.7)	0.457
Yes	48	15.6	7 (12.5)	41 (16.3)	
Ever enrolled in a Drug Addiction treatment program					
Never been treated	263	85.7	51 (91.0)	212 (84.5)	0.395
Ever been treated	21	6.8	3 (5.4)	18 (7.2)	
Refused to answer	23	7.5	2 (3.6)	21 (8.3)	

Bold values are for variables that were found to be statistically significantly different between male and female PWID

* Data missing for n=2 ; ** Data missing for n=9 ; *** Data missing for n=1 ; **** Data missing for n=3

### Analysis

We calculated crude estimates, including means and proportions, for sociodemographic characteristics, the outcomes of interest, and other covariates. Pearson’s Chi-squared tests (*χ*^2^) were used to compare demographic and IDU behaviors by biological sex, and an alpha level of 0.05 was used to attribute statistical significance.

For both outcomes, we applied Lowess smoothed nonparametric regressions to choose appropriate scales for age and duration of injection drug use. For HIV infection, both variables showed an approximately linear association and were analyzed as continuous variables. However, they were not linearly associated with recent needle sharing and thus were analyzed as categorical variables in the models for the second outcome. All other variables were analyzed as binary or categorical variables for both outcomes (Tables 3 and 4).

**Table 3:** Factors associated with HIV infection among PWID in Kigali, Rwanda, *N*: 307

	N Living with HIV	% Living with HIV	Unadjusted PR (95% CI)	*p* value	Adjusted PR (95% CI)	*p* value
Sociodemographic characteristics						
Age in years*			**1.05 (1.01–1.09)**	**0.02**	0.98 (0.91–1.07)	0.842
Biological sex						
Female	8	14.3	Ref			
Male	21	8.4	0.58 (0.27–1.25)	0.169	1.86 (0.86–4.04)	0.118
Education						
Primary education or less	7	14.9	Ref		Ref	
Some secondary education	3	3.8	**0.25 (0.07–0.94)**	**0.04**	0.29 (0.09–1.04)	0.06
Completed secondary or above	19	10.5	0.71 (0.31–1.57)	0.395	0.64 (0.27–1.39)	0.239
Marital status**						
Single	20	7.9	Ref		Ref	
Cohabitating/Married	2	6.1	0.76 (0.18–3.13)	0.708	0.75 (0.21–2.74)	0.67
Divorced/Separated/Widow	7	36.8	**4.64 (2.25–9.58)**	**0.001**	**3.86 (1.49–10.01)**	**0.005**
Occupation						
Unemployed	15	8.9	Ref			
Employed	14	10.9	1.23 (0.62–2.46)	0.554		
Self-reported sexual orientation***						
Heterosexual	17	7.9	Ref			
Homosexual	4	11.4	1.44 (0.52–4.05)	0.484		
Bisexual	8	14.3	1.81 (0.82–3.97)	0.141		
*Drug Injection related behaviors, access to needles and condom use*						
Age at first injection						
Before 18 years	5	9.4	Ref			
18 to 24 years	12	9.1	1.36 (0.57–3.23)	0.48		
Over 25 years	12	9.8	1.82 (0.75–4.37)	0.183		
Duration of drug injection****						
Years injecting drugs			**1.06 (0.99–1.11)**	**0.055**	1.04 (0.92–1.18)	0.494
Needle sharing in the previous 6 months						
Never	15	7.1	Ref		Ref	
Rarely	6	10	1.42 (0.57–3.51)	0.447	1.31 (0.44–3.93)	0.625
Half the time or more	8	23.5	**3.34 (1.53–7.28)**	**0.002**	**2.77 (1.28–5.98)**	**0.009**
Number of needles sharing partners in the previous 6 months						
None	7	11.1	Ref			
One to two	15	8.2	0.74 (0.31–1.73)	0.484		
Three or more	7	15.9	1.43 (0.54–3.79)	0.471		
Selling sex for drugs in the previous 6 months*****						
No	16	7.8	Ref			
Yes	13	13.8	1.76 (0.88–3.52)	0.108		
Access to sterile needles						
Always	20	10.2	Ref			
Sometimes	9	8.9	0.87 (0.41–1.85)	0.723		
Condom use						
Always	10	9.2	Ref			
Never or sometimes	19	9.6	1.04 (0.50–2.17)	0.904		

Bold values are for variables that were found to be significantly associated with prevalent HIV infection

* Data missing for n=2; ** Data missing for n=3; *** Data missing for n=1 ; **** Data missing for n=2 ; ***** Data missing for n=9

**Table 4:** Factors associated with needle sharing in the six months preceding the study among PWID in Kigali, Rwanda, *N*: 307

	Shared needles % (*N*)	Unadjusted PR (95% CI)	*p *value	Adjusted PR (95% CI)	*p* value
Sociodemographic characteristics*					
18–24	22.9 (19)	Ref		Ref	
25–34	32.9 (62)	1.44 (0.92–2.25)	0.108	1.01 (0.71–1.42)	0.978
> 35	38.2 (13)	1.67 (0.93–2.99)	0.084	0.91 (0.57–1.45)	0.698
Biological sex					
Male	30.7 (77)	Ref		Ref	
Female	30.4 (17)	0.98 (0.64–1.53)	0.963	**1.68 (1.09–2.59)**	**0.019**
Education					
Primary education or less	23.4 (11)	Ref			
Some secondary education	21.5 (17)	0 .92 (0.47–1.79)	0.805		
Completed secondary or above	36.5 (66)	1.56 (0.89–2.71)	0.116		
Marital status**					
Single	29.7 (75)	Ref			
Cohabitating/Married	33.3 (11)	1.12 (0.66–1.88)	0.669		
Divorced/Separated/Widow	36.8 (7)	1.24 (0.66–2.31)	0.501		
Occupation					
Unemployed	25.4 (43)	Ref		Ref	
Employed/ Student	36.9 (51)	**1.45 (1.03–2.04)**	**0.031**	**1.58 (1.19–2.11)**	**0.002**
Self-reported sexual orientation***					
Heterosexual	27.4 (59)	Ref		Ref	
Homosexual	28.6 (10)	1.04 (0.58–1.84)	0.889	1.08 (0.75–1.55)	0.667
Bisexual	44.6 (25)	**1.63 (1.13–2.34)**	**0.009**	**1.46 (1.03- 2.07)**	**0.034**
*Drug Injection related behaviors, access to needles and condom use*					
Age at first injection					
Before 18 years	30.2 (16)	Ref			
18 to 24 years	33.3 (44)	1.10 (0.68–1.78)	0.683		
Over 25 years	27.9 (34)	0.92 (0.56–1.52)	0.754		
Duration of drug injection****					
Less than 3 years	20.3 (26)	Ref		Ref	
4–5 years	30.5 (32)	**1.50 (0.95–2.35)**	**0.077**	1.05 (0.74–1.48)	0.791
Over 5 years	50.7 (36)	**2.49 (1.65–3.77)**	**0.001**	1.25 (0.83–1.89)	0.281
Needle sharing at the first injection					
No	15.4 (36)	Ref		Ref	
Yes	79.5 (58)	**5.16 (3.73–7.13)**	**0.001**	**2.18 (1.59–2.99)**	**0.001**
Needle reuse in the previous 6 months					
No	9.8 (20)	Ref		Ref	
Yes	71.8 (74)	**7.32 (4.74–11.31)**	**0.001**	**2.27 (1.51–3.43)**	**0.001**
Number of needles sharing partners in the previous 6 months					
Two or less	21.9 (54)	Ref		Ref	
More than two partners	90.9 (40)	**4.14 (3.21–5.34)**	**0.001**	0.99 (0.79–1.25)	0.983
Selling sex for drugs in the previous 6 months*****					
No	24 (49)	Ref		Ref	
Yes	42.5 (40)	**1.77 (1.26–2.48)**	**0.001**	1.08 (0.79–1.46)	0.607
Access to sterile needles					
Always	19.4 (38)	Ref		Ref	
Sometimes	52.5 (53)	**2.71 (1.92–3.81)**	**0.001**	1.10 (0.84–1.44)	0.477
Sharing other drug paraphernalia in the previous 6 months					
Always	19.4 (38)	Ref		Ref	
Sometimes	52.5 (53)	**9.38 (6.19–14.21)**	**0.001**	**3.56 (2.19- 5.81)**	**0.001**

Bold values are for variables that were found to be significantly associated with needle sharing in the six months prior to the study

* Data missing for n=2; ** Data missing for n=3; *** Data missing for n=1 ; **** Data missing for n=2 ; ***** Data missing for n=9

Bivariable Poisson regression models with robust variance estimation were fitted to compute prevalence ratios (PR) and 95% confidence intervals (CI) determining the association between sociodemographic factors and IDU practices for both outcomes. Poisson regressions were used because both outcomes were common and log binomial models failed to converge. The final models for both outcomes were constructed using variables that were associated with the outcome if *p* < 0.1 in the bivariable analyses. Sex and age were included in the final multivariable models irrespective of their associations with the outcomes in the bivariable analyses. Analyses were performed with Stata version 14.2 (StataCorp, College Station, TX) statistical package.

## Results

### Sociodemographic characteristics and HIV infection

Overall, 322 PWID were recruited, but analyses were restricted to 307 participants for whom HIV status information was available (five participants refused testing and 10 were missing testing results). The median age of participants was 28 years (IQR:24–31), and 81% (248) were males (Table 1).

The prevalence of HIV in this group was 9.5% (95% CI 8.7–9.3).

### Injection drug use history and practices

Median age at first injection was 23 (IQR 20–27), but 17% (53) of participants had their first injection before reaching 18. The majority of participants, 57% (176), had been injecting for four or more years. Nearly all, 99% (304), injected heroin their first time.

In the six months preceding the study, heroin was the primary drug of choice for 99% (303) of participants. However, 10% (31) and 4% (12) also reported injecting cocaine and methamphetamine, respectively. Many participants, 30% (93), had used a drug combination in the six months before the study. The most common combination of drugs was heroin and marijuana, reported by 23% (70) of participants, while alcohol use was reported in combination with heroin by 9% (28). IDU frequency was high with 95% (293) of participants reporting at least one injection per day in the previous six months.

Overall, 31% (94) of participants reported recent needle sharing. However, up to 91% (280) of participants reported ever sharing needles in their lifetimes. Furthermore, 33% (103) reported reusing needles in the previous six months. Most participants, 98% (301), reported getting sterile syringes and needles from pharmacies and 20% (61) reported also getting needles from their networks, including friends and sex partners. However, 34% (101) of participants reported inconsistent access to sterile needles for injection purposes.

There were several differences in IDU practices between males and females. Females were more likely to have been injected by a sex partner the first time (*χ*^2^ *p* < 0.007), to have recently initiated IDU (*χ*^2^ *p* < 0.007), and to report selling sex for drugs compared to males (*χ*^2^ *p* < 0.0001) (Table 2).

Regarding HIV-related knowledge and behavior, 87% (268) reported knowing that IDU was a risk factor for HIV infection. Over half of participants, 65% (198), reported inconsistent condom use in the previous six months.

### Factors associated with HIV infection

In the final multivariable model, PWID who reported sharing needles half the time or more in the six months prior to the study were more likely to be living with HIV compared to those who did not share needles (adjusted prevalence ratio (aPR) 2.67; 95% CI 1.23–5.78) (Table 3).

### Factors associated with recent needle sharing

In the multivariable analyses, several demographic and IDU variables were associated with recent needle sharing. Female PWID were more likely to share needles during drug injection compared to male PWID (aPR 1.68; 95% CI 1.09–2.58). Additionally, bisexual individuals (aPR 1.48; 95% CI 1.03–2.07) were more likely than heterosexual individuals to share needles.

Regarding IDU history and practices, participants who had shared needles during their first injection were more likely to have shared needles recently (aPR 2.18; 95% CI 1.58–2.99). Furthermore, needle reuse (aPR 2.27; 95% CI 1.51–3.43) and sharing other drug injection equipment (aPR 3.56; 95% CI 2.19–5.81) were positively associated with needle sharing (Table 4).

## Discussion

This study is one of the first to characterize the population of PWID in Kigali and to describe the burden of HIV in this community. Moreover, it clearly demonstrates the existence of individual risk factors in this population that are known to be associated with HIV, including needle sharing and inconsistent condom use. These practices highlight substantial potential risks of onward transmission and acquisition of HIV and other blood-borne infections among PWID. These results show an urgent need for implementation of evidence-based harm reduction strategies and other individual-, network-, and structural-level interventions to reduce morbidity and mortality among PWID in Rwanda.

In this convenience-based sample, PWID carried a disproportionate burden of HIV over three times what is observed in the general population [15]. These findings are consistent with other African studies, underscoring the need to address the HIV prevention and treatment needs of PWID [1]. However, some countries have reported higher HIV burden among PWID (e.g., Kenya 14–20%; Mozambique 20–50%), likely reflecting the overall higher burden of HIV in these countries compared to Rwanda [16–19]. Injection partners with whom one shares injection equipment often serve as a primary mode of HIV acquisition among PWID. However, risk of HIV and OBBI among PWID can be further compounded by sexual risk behaviors (e.g., condomless sex). Our results suggest that consistent condom use in this population is low, which is expected given that research suggests riskier sexual practices are associated with substance abuse [20]. Globally, studies indicate that sexual risks are elevated among PWID in countries across SSA relative to other regions, highlighting the importance of understanding the intersecting impact of sexual and injection drug risks among PWID in Rwanda [1]. Implementing sexual health programs, like condom distribution and HIV/STI management, could mitigate the added sexual risks that PWID face. Successes of the Rwandan HIV program in the general adult population could be leveraged to develop initiatives tailored to PWID needs.

Importantly, we observed a high prevalence of risky IDU practices. A high proportion of participants reported reusing needles, which is known to be associated with severe infections including abscesses, septicemia, and infective endocarditis [21–23]. We also found that nine out of ten participants reported ever sharing needles, while over one-third reported sharing needles in the prior 6 months. Consistent with existing literature, needle sharing was positively associated with HIV infection in this study [16, 17, 19]. Taken together, these data demonstrate the urgency for implementing comprehensive harm reduction interventions in Rwanda, particularly syringe service programs (SSP), to provide sterile injection equipment and education on safer injection techniques [24]. Through provision of sterile needles and other drug paraphernalia, SSPs have been shown to effectively reduce unsafe injection practices and injection frequency, to facilitate linkage to substance use disorder treatment programs including medications for opioid use disorder (MOUD) programs, and to be cost-effective [25–27]. Notably, we have found that female PWID, bisexual individuals, PWID who shared needles during their first injection, those who reused needles recently, and those that share other drug injection paraphernalia had higher proportions of needle sharing compared to others. Consequently, careful consideration of these factors will be needed for optimal SSPs in Rwanda.

In addition to SSPs, there is a necessity for overdose prevention and treatment of substance use disorders in Rwanda. Despite naloxone already being available in some pharmacies in Rwanda [28], nearly half of participants reported knowing someone who died from a drug-related overdose. This calls for immediate introduction of overdose prevention and treatment programs and strategies to optimize distribution and use of naloxone in Kigali. Community-based overdose prevention and response programs have been implemented in other settings, and there is evidence that these programs can save lives [29–31]. Finally, over half of participants reported wanting to reduce or quit drug injection practices; however, only 15% reported ever attending a substance use treatment program, highlighting a need for increased coverage and availability of programs such as MOUD. Heroin was the primary drug of choice, as observed in other countries in the region [2]. Additionally, some participants reported injecting cocaine and methamphetamine. It is possible that there are more drug types being used in Kigali that are not reported in this study, most likely because participants were not asked about them during interviews. Given the high frequency of injection reported (i.e., most participants injected daily) and the likely high number of overdoses, a comprehensive study evaluating the types of drugs available in Kigali and their chemical composition is needed to inform programming for PWID in Kigali.

Another contribution of this study is characterizing the demographic characteristics of PWID in Kigali by age, sex assigned at birth, and sexual orientation. Almost one-fifth of participants reported their first injection before age 18. The 2020 World Drug Report highlighted the growing demand for injection drugs, particularly among young adults across African countries [2, 32]. This is evidenced by the fact that almost half of participants in our study reported injecting drugs for fewer than 3 years. This was especially the case for female PWID who were more likely to have recently initiated IDU. Additionally, although there was no statistical association between biological sex and HIV infection, female PWID in our study reported a higher prevalence of selling sex for drugs consistent with other studies among female PWID [33, 34]. PWID who also report sex work are at a higher risk of HIV acquisition compared to other PWID [7]. Furthermore, one in three female PWID was injected by a sexual partner the first time they injected a drug, which demonstrates the complex interplay of sexual and IDU practices. Sex differences in the risk of HIV and other infectious diseases acquisition among PWID have been documented in other settings [34, 35]. Finally, these data also show that sexual orientation is an important consideration in PWID programming in Rwanda. One in ten PWID and nearly one in five self-identified as MSM and bisexual, respectively. Members of sexual minority groups are known to be at a higher risk for both intersecting stigmas as well as the acquisition of HIV and other STIs in Rwanda [12]. Thus, there is a need for further studies detailing social and health experiences of different demographic subgroups of PWID including female PWID and sexual and gender minorities who use drugs. PWID programs should also be cognizant of the complex interplay of sexual identities and sexual and IDU practices to offer optimal interventions to all members of KP groups.

Our study has several limitations. First, our sample is convenience-based. We are therefore unable to make inferences to the larger PWID population in Kigali or Rwanda. Furthermore, the cross-sectional nature of the study limits our ability to make temporal inferences between the variables of interest and HIV infection. Second, separate from HIV testing, our measurers are self-reported and may be subject to recall or social desirability bias. However, we observed high prevalence of injection and needle sharing behaviors, thus providing a benchmark with which to inform programmatic planning for PWID in Rwanda. Our study was also limited in that it did not collect information on the behaviors of injection partners. The importance of injection drug networks on the transmission and acquisition of blood-borne infections, including HIV, and the social diffusion of behaviors and other network norms, have been well documented in other settings [36]. Our finding that half of participants reported injecting with someone else at first injection, indicates the potential for implementing network-based interventions to reach PWID with services and educational messages early in their injection history. Finally, while the study identifies a proportion of the sample that are aware of their HIV status, we are unable to determine HIV outcomes further downstream of the HIV care continuum, including antiretroviral uptake and viral suppression. In particular, viral suppression could serve as a marker to gauge the success of HIV treatment programs in this population. The lack of HBV and HCV testing in this study presents another limitation given the high burden of these infections among PWID globally [1]. Despite these limitations, our study provides critical information to understand the HIV programming needs of PWID in Kigali.

Future research should leverage respondent-driven sampling, or other sampling approaches used to reach and estimate the size of historically hidden populations without a known sampling frame [37, 38]. Stronger sampling approaches could facilitate data collection for larger epidemiological studies, enabling estimation of the prevalence of HIV, HBV, HCV, and other health (e.g., viral suppression) and social outcomes (e.g., mental health), as well as the impact of structural factors (e.g., stigma, criminalization) on these outcomes at the community or population level. Estimating the size of the PWID population would allow for better programmatic planning to address the HIV and other health needs of PWID in Rwanda.

## Conclusion

The findings of this study call for immediate action. PWID have a high prevalence of HIV and self-reported injection practices associated with substantial onward risk of transmission and acquisition of HIV and other blood-borne infections. Implementation of evidence-based comprehensive harm reduction programs is not only a public health emergency but also a human rights and moral imperative. As of 2021, few African countries have adopted syringe services programs, and even fewer have government-led comprehensive harm reduction services in place. The data presented here suggest that Rwanda should join this list to save lives of people who use drugs and building the evidence base to support implementation across the African continent.

## Data Availability

The data are owned by the partner institutions. Requests for data utilization should be sent to Aflodis Kagaba at: kagaba@hdirwanda.org.
