# Correction: Injection drug use practices and HIV infection among people who inject drugs in Kigali, Rwanda: a cross-sectional study

**DOI:** 10.1186/s12954-022-00674-w

**Published:** 2022-08-18

**Authors:** Jean Olivier Twahirwa Rwema, Vianney Nizeyimana, Neia M. Prata, Nneoma E. Okonkwo, Amelia A. Mazzei, Sulemani Muhirwa, Athanase Rukundo, Lisa Lucas, Audace Niyigena, Jean Damascene Makuza, Chris Beyrer, Stefan D. Baral, Aflodis Kagaba

**Affiliations:** 1 Department of Epidemiology, Key Populations Program, Center for Public Health and Human Rights, Johns Hopkins Bloomberg School of Public Health, 615 N Wolfe Street E 7133, Baltimore, MD 21205 USA; 2 Health Development Initiative, Kigali, Rwanda; 3 School of Medicine, Johns Hopkins University, Baltimore, MD USA; 4 Emory University School of Medicine, Atlanta, GA USA; 5 Projet San Francisco, Kigali, Rwanda; 6 Département de Psychiatrie, Service d’addictologie, Hôpitaux Universitaire de Genève, Geneva, Switzerland; 7 School of Population and Public Health, University of British Columbia, Vancouver, BC Canada; 8 BC Center for Disease Control, Vancouver, BC Canada; 9 Rwanda Biomedical Center, Kigali, Rwanda

## Correction to: Harm Reduction Journal (2021) 18:130 10.1186/s12954-021-00579-0

Following the publication of the original article [1], There is a typo error, under Results heading.

Incorrect text is:

The prevalence of HIV in this group was 9.5% (95% CI 8.7–9.3).

Correct text is:

The prevalence of HIV in this group was 9.5% (95% CI 6.6–13.3).

Also in Table 4, there is an error on the label of the categories of the variable named: “sharing other drug paraphernalia in the previous 6 months”. The correct table is shown below:

**Table 4:** Factors associated with needle sharing in the six months preceding the study among PWID in Kigali, Rwanda, N:307

	Shared needles % (*N*)	Unadjusted PR (95% CI)	*p* value	Adjusted PR (95% CI)	*p* value
Sociodemographic characteristics					
18–24	22.9 (19)	Ref		Ref	
25–34	32.9 (62)	1.44 (0.92–2.25)	0.108	1.01(0.71–1.42)	0.978
> 35	38.2 (13)	1.67 (0.93–2.99)	0.084	0.91(0.57–1.45)	0.698
Biological sex					
Male	30.7 (77)	Ref		Ref	
Female	30.4 (17)	0.98 (0.64–1.53)	0.963	**1.68(1.09–2.59)**	**0.019**
Education					
Primary education or less	23.4 (11)	Ref			
Some secondary education	21.5 (17)	0 0.92 (0.47–1.79)	0.805		
Completed secondary or above	36.5 (66)	1.56 (0.89–2.71)	0.116		
Marital status					
Single	29.7 (75)	Ref			
Cohabitating/married	33.3 (11)	1.12 (0 0.66–1.88)	0.669		
Divorced/separated/widow	36.8 (7)	1.24 (0.66–2.31)	0.501		
Occupation*					
Unemployed	25.4 (43)	Ref		Ref	
Employed/student	36.9 (51)	**1.45 (1.03–2.04)**	**0.031**	**1.58 (1.19–2.11)**	**0.002**
Self-reported sexual orientation					
Heterosexual	27.4 (59)	Ref		Ref	
Homosexual	28.6 (10)	1.04 (0.58–1.84)	0.889	1.08 (0.75–1.55)	0.667
Bisexual	44.6 (25)	**1.63 (1.13–2.34)**	**0.009**	**1.46 (1.03–2.07)**	**0.034**
*Drug injection related behaviors, access to needles and condom use*					
Age at first injection					
Before 18 years	30.2 (16)	Ref			
18–24 years	33.3 (44)	1.10 (0.68–1.78)	0.683		
Over 25 years	27.9 (34)	0.92 (0.56–1.52)	0.754		
Duration of drug injection					
Less than 3 years	20.3 (26)	Ref		Ref	
4–5 years	30.5 (32)	**1.50 (0.95–2.35)**	**0.077**	1.05 (0.74–1.48)	0.791
Over 5 years	50.7 *36)	**2.49 (1.65–3.77)**	**0.001**	1.25 (0.83–1.89)	0.281
Needle sharing at the first injection					
No	15.4 (36)	Ref		Ref	
Yes	79.5 (58)	**5.16 (3.73–7.13)**	**0.001**	**2.18 (1.59–2.99)**	**0.001**
Needle reuse in the previous 6 months					
No	9.8 (20)	Ref		Ref	
Yes	71.8 (74)	**7.32 (4.74–11.31)**	**0.001**	**2.27 (1.51–3.43)**	**0.001**
Number of needles sharing partners in the previous 6 months					
Two or less	21.9 (54)	Ref		Ref	
More than two partners	90.9 (40)	**4.14 (3.21–5.34)**	**0.001**	0.99 (0.79–1.25)	0.983
Selling sex for drugs in the previous 6 months					
No	24 (49)	Ref		Ref	
Yes	42.5 (40)	**1.77 (1.26–2.48)**	**0.001**	1.08 (0.79–1.46)	0.607
Access to sterile needles					
Always	19.4 (38)	Ref		Ref	
Sometimes	52.5 (53)	**2.71 (1.92–3.81)**	**0.001**	1.10 (0.84–1.44)	0.477
Sharing other drug paraphernalia in the previous 6 months					
No	19.4 (38)	Ref		Ref	
Yes	52.5 (53)	**9.38 (6.19–14.21)**	**0.001**	**3.56 (2.19–5.81)**	**0.001**

Bold values are for variables that were found to be significantly associated with needle sharing in the six months prior to the study

*Data missing for *n* = 2; **Data missing for *n* = 3; *** Data missing for *n* = 1; **** Data missing for *n* = 2; *****Data missing for *n* = 9

The original article has been corrected.

